# Genomic Epidemiology and Characterization of Carbapenem-Resistant Klebsiella pneumoniae in ICU Inpatients in Henan Province, China: a Multicenter Cross-Sectional Study

**DOI:** 10.1128/spectrum.04197-22

**Published:** 2023-05-22

**Authors:** Shanmei Wang, Lei Wang, Jing Jin, Gang Li, Huanzhang Shao, Yang Song, Yuanzheng Sun, Yan Zhang, Jianjian Cheng, Lifeng Li

**Affiliations:** a Department of Clinical Microbiology, Henan Provincial People's Hospital, Zhengzhou University People's Hospital, Henan University People's Hospital, Zhengzhou, Henan, China; b Department of Bioinformatics Research, Genskey Co., Ltd., Beijing, China; c Department of Pathogen Biology and Immunology, Henan Medical College, Zhengzhou, Henan, China; d Department of Critical Care Medicine, Henan Provincial People's Hospital, Zhengzhou University People's Hospital, Henan University People's Hospital, Zhengzhou, Henan, China; e Clinical Laboratory, Yuzhou Jundu Hospital, Xuchang, Henan, China; f Clinical Laboratory, Yima People’s Hospital, Sanmenxia, Henan, China; g Department of Respiratory and Critical Care Medicine, Henan Provincial People's Hospital, Zhengzhou University People's Hospital, Henan University People's Hospital, Zhengzhou, Henan, China; University of L'Aquila

**Keywords:** genomic surveillance, molecular epidemiology, *Klebsiella pneumoniae*, carbapenem resistance, sequence typing, MDR, XDR, hypermucoviscosity

## Abstract

Carbapenem-resistant Klebsiella pneumoniae (CRKP) has disseminated globally and is difficult to treat, causing increased morbidity and mortality rates in critically ill patients. We conducted a multicenter cross-sectional study of intensive care unit (ICU) inpatients in 78 hospitals to investigate the prevalence and molecular characteristics of CRKP in Henan Province, China, a hyperepidemic region. A total of 327 isolates were collected and downsampled to 189 for whole-genome sequencing. Molecular typing revealed that sequence type 11 (ST11) of clonal group 258 (CG258) was predominant (88.9%, *n* = 168), followed by ST2237 (5.8%, *n* = 11) and ST15 (2.6%, *n* = 5). We used core genome multilocus sequence typing (cgMLST) to further classified the population into 13 subtypes. Capsule polysaccharide (K-antigen) and lipopolysaccharide (LPS; O-antigen) typing revealed that K64 (48.1%, *n* = 91) and O2a (49.2%, *n* = 93) were the most common. We studied isolates collected from both the airway and the gut of the same patients and showed that intestinal carriage was associated with respiratory colonization (odds ratio = 10.80, *P* < 0.0001). Most isolates (95.2%, *n* = 180) showed multiple drug resistance (MDR), while 59.8% (*n* = 113) exhibited extensive drug resistance (XDR), and all isolates harbored either *bla*_KPC-2_ (98.9%, *n* = 187) or *bla*_CTX-M_ and *bla*_SHV_ extended-spectrum beta-lactamases (ESBLs) (75.7%, *n* = 143). However, most were susceptible to ceftazidime-avibactam (CZA) (94.7%, *n* = 179) and colistin (97.9%, *n* = 185). We found *mgrB* truncations in isolates conferring resistance to colistin and mutations in *bla*_SHV_ and OmpK35 and OmpK36 osmoporins in CZA-resistant isolates. Using a regularized regression model, we found that the aerobactin sequence type and the salmochelin sequence type, among others, were predictors of the hypermucoviscosity phenotype.

**IMPORTANCE** In this study, we address the ongoing epidemic of carbapenem-resistant Klebsiella pneumoniae, a critical threat to public health. The alarming genotypic and phenotypic convergence of multidrug resistance and virulence highlights the increasingly aggravated threat posed by K. pneumoniae. This calls for a combined effort of physicians and scientists to study the potential mechanisms and establish guidelines for antimicrobial therapies and interventions. To this end, we have conducted a genomic epidemiology and characterization study using isolates collected in a coordinated effort of multiple hospitals. Innovative biological discoveries of clinical importance are made and brought to the attention of clinical researchers and practitioners. This study presents an important advance in the application of genomics and statistics to recognize, understand, and control an infectious disease of concern.

## INTRODUCTION

Klebsiella pneumoniae is one of the most common opportunistic bacteria that can cause life-threatening infections, including pneumonia, meningitis, necrotizing fasciitis, endophthalmitis, liver abscess, and bloodstream infections (1, 2). Sounding the alarm of global public health, carbapenem-resistant Klebsiella pneumoniae (CRKP) has been isolated at an increasing rate (3). CRKP is particularly dangerous because therapeutic options are limited and carbapenems are considered one of the last resorts in the ammunition of clinicians (4, 5). In recent molecular and clinical epidemiological surveys like EuSCAPE and CRACKLE-2, CRKP was identified as the most common carbapenemase-producing *Enterobacteriales*, with high resistance to last-line antibiotics (6–8). Henan is a hot spot region of the CRKP epidemic in China. According to statistics from China Antimicrobial Resistance Surveillance System (CARSS), Henan was among the provinces with the highest incidence rate of K. pneumoniae, and the isolates in Henan had the highest carbapenem resistance rate (32.8%) (9). Here, we aim to study the epidemiology and genomic characteristics of CRKP in Henan by making use of whole-genome sequencing (WGS), multilocus sequence typing (MLST), and core genome multilocus sequence typing (cgMLST) (10, 11). With cgMLST, heterogeneous isolates of the same sequence type (ST) can be further classified, and linkage in clonal transmission can be inferred, as demonstrated previously (12). With whole-genome sequence data, it is also possible to identify and type outer membrane polysaccharide capsule antigen (K) and lipopolysaccharide (LPS; O) loci and thus predict clinically relevant antigen types (13).

Nosocomial infection and in-hospital outbreaks of CRKP are common, owing to its ability to spread rapidly in the hospital environment (14, 15). The use of ventilators and intravenous catheters in intensive care units (ICUs) is among the common risk factors for nosocomial infections of K. pneumoniae (16). Nevertheless, it has been reported that high admission prevalence, rather than patient-to-patient transmission, is the main factor driving endemicity (17). Gastrointestinal carriage is a key risk factor for nosocomial K. pneumoniae infection, associated with a 4-fold increase of risk in intensive care and oncology patients (18). Clinical typing of K. pneumoniae that commensally colonizes the gut and the respiratory tract suggests that gastrointestinal carriage is a major reservoir that accounted for infections in intensive care patients (19). The duration of gut colonization can exceed 12 months (20, 21). Infections can subsequently originate from the gastrointestinal reservoir by penetration of the intestinal epithelium via a transcellular pathway (15, 22).

Carbapenem resistance in CRKP is often caused by the production of beta-lactamases such as *bla*_KPC_ and *bla*_NDM_ (23), and its treatment usually involves non-beta-lactam antibiotics and beta-lactamase inhibitors, such as fosfomycins, polymyxins, fluoroquinolones, tetracyclines, sulfamethoxazole-trimethoprim (SXT), and ceftazidime-avibactam (CZA) (24, 25). However, these alternative therapeutic options come with concerns regarding their elevated toxicity and limited efficacy due to cross-resistance (26, 27). A better understanding of the molecular basis of antimicrobial resistance (AMR) is needed to optimize their usage.

The recent emergence of community-acquired infections caused by hypervirulent Klebsiella pneumoniae (hvKP) brings additional challenges to the global health issue caused by K. pneumoniae (2). The hvKP can be identified by its hypermucoviscosity phenotype in the string test and is more invasive and pathogenic (2, 28). It is important to identify biomarkers to differentiate hvKP and classical K. pneumoniae, and many effects have been made to this end (29–31). However, it was found in a recent study that genetic markers were not correlated well with the hypervirulent phenotypes, suggesting that there is room for improvement in the marker identification methods (32). In addition, the convergence of antimicrobial resistance and hypervirulence from historically nonoverlapping subpopulations poses greater challenges to clinicians (33–36). There is evidence suggesting that CRKP strains acquiring virulence plasmids are more suitable for survival in hospital settings than strains that evolved along other paths (37).

## RESULTS

### The collection of CRKP isolates and their genomic characteristics.

A total of 327 carbapenem-resistant K. pneumoniae isolates were recovered from 1,058 ICU inpatients in 78 participating hospitals in Henan Province on 10 March 2021. The origins of the specimens are summarized in Table S1 in the supplemental material. The total prevalence of CRKP per ICU patient, including from all types of specimens, was 20.9% (*n* = 228), and the prevalence in each city is summarized in Table S2. We performed WGS on 189 of the isolates and obtained 9.30 million mean clean reads per isolate, giving an average depth of ~250×. The assembled genomes had a mean length of 5.770 Mbp (range, 5.408 Mbp to 6.194 Mbp) and consisted of 124.3 contigs on average. The GC content of the assemblies ranged from 56.18% to 57.39%, with a mean of 56.98%. Completeness and contamination rates were >97.02% and <1.58%, respectively (Fig. S1 and Table S3). Genomic feature annotation identified, on average, 5,494.0 protein-coding sequences and 5.8 rRNA and 70.0 tRNA copies per genome (Table S4). The pan-genome of all the isolates in this collection contained 3,569 core genes. The rarefaction curve confirmed that the size of the sample we recruited was large enough to define the core genome of this subpopulation (Fig. S2). By comparing the Mash score against reference assemblies, one of the isolates, KP211G, was identified as Klebsiella quasipneumoniae subsp. *similipneumoniae*, also known as the KpII-b phylogroup (38). It had initially been misidentified as K. pneumoniae, for matrix-assisted laser desorption ionization–time of flight mass spectrometry (MALDI-TOF MS) could not identify species within the K. pneumoniae *sensu lato* complex with sufficient accuracy (39).

### Molecular epidemiology.

**FIG 1:**
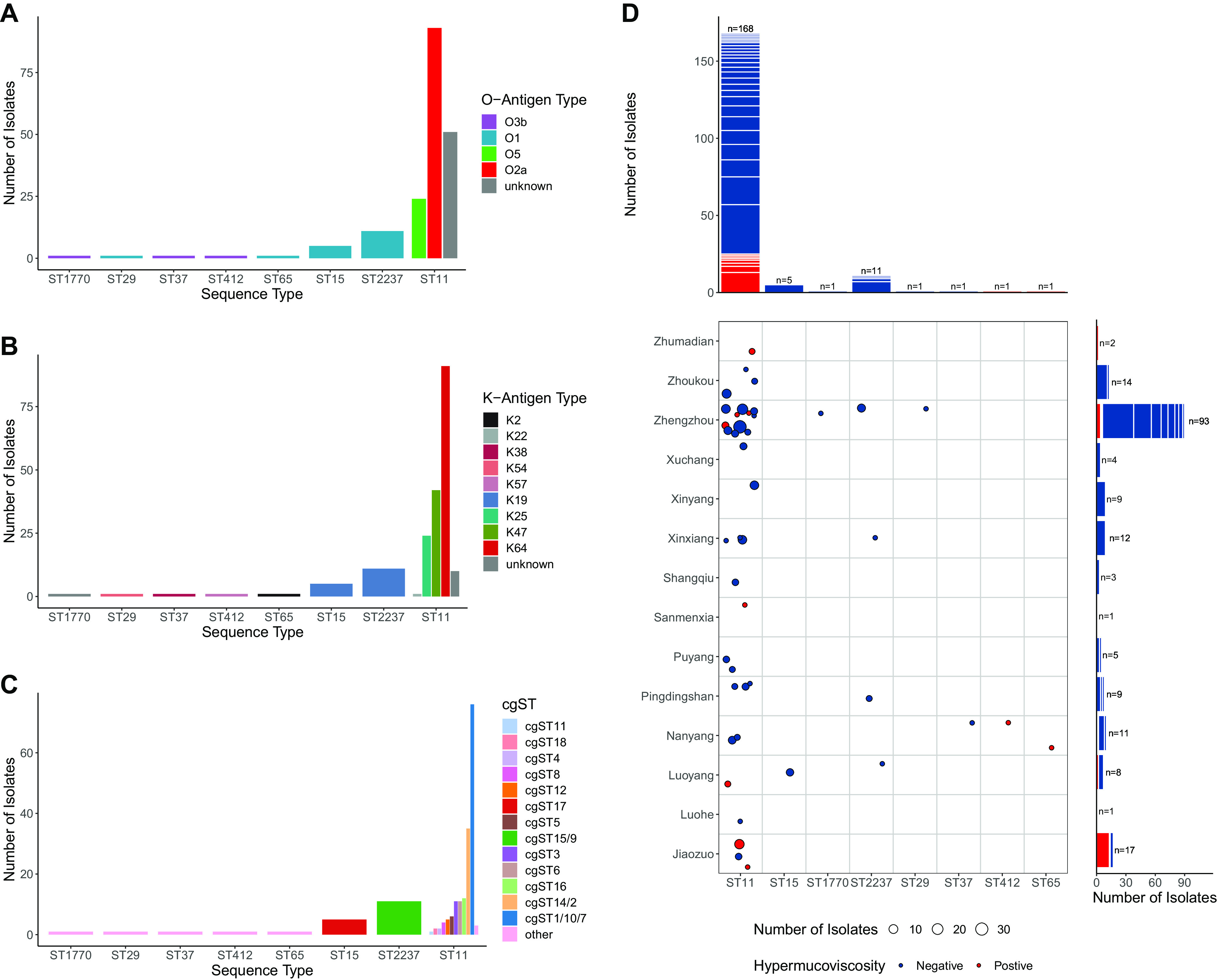
Molecular typing. (A) Distribution of K-antigen types in each of the ST lineages. (B) O-antigen types. (C) Breakdown of core genome sequence types (cgSTs) in each of the ST lineages. (D) Distribution of cgSTs. Each circle represents a cgST, scaled by the number of genomes in the cluster, and colored red if more than half of the isolates showed hypermucoviscosity. Each block in the marginal bar plots indicates a cgST group, and the total height shows the number of isolates in each geographic region (right) or ST lineage (top).

*In silico* 7-gene MLST identified 9 sequence types in the collection. ST11 was the most prevalent type in our collection of CPKP, making up 88.9% (*n* = 168) of the isolates. It was followed by ST2237 (5.8%, *n* = 11) and ST15 (2.6%, *n* = 5), and other sequence types were ST29, ST37, ST65, ST412, and ST587, with one isolate for each of them. The only K. quasipneumoniae isolate was of type ST1770. ST11 is a single-locus variant of ST258, and they both belong to the well-studied clonal group 258 (CG258). ST15 belongs to CG15 (including ST14 and ST15), while ST65 belongs to CG65 (including ST65 and ST375). ST29, ST37, and ST412 are relatively uncommon singletons that have seen few reports. It is noteworthy that ST2237 is a recently identified sequence type that has only been reported in Henan, at a frequency of 5% (40). ST587 is a new sequence type of K. pneumoniae that has not been reported. In the cgMLST analysis, the isolates were further classified at a higher resolution. Fourteen single-linkage clusters were found, based on the allelic differences in their core genome. The clusters were assigned a stable nomenclature, allowing comparison with the results of other epidemiological studies. The majority of the isolates belonging to ST11 were classified as cgST1, cgST10, and cgST7 (45.2%, *n* = 76); cgST14 and cgST2 (20.8%, *n* = 35); cgST16 (7.1%, *n* = 12); cgST3 (6.5%, *n* = 11); and cgST6 (6.5%, *n* = 11). ST15 isolates were all cgST17 (*n* = 5), while ST2237 isolates all belonged to cgST15 and cgST9 (*n* = 11) (Fig. 1; Table S5).

According to the prediction based on gene locus sequences, 9 capsular types (K-antigen) were identified in our collection. The most common ones were K64 (48.1%, *n* = 91), K47 (22.2%, *n* = 42), K25 (12.7%, *n* = 24), and K19 (8.5%, *n* = 16). In ST11 isolates, 54.2% (*n* = 91) were of K64, 25.0% (*n* = 42) were K47, and 14.3% (*n* = 24) were K25, while all of the ST15 and ST2237 isolates were classified as K19. O-antigen typing identified 4 lipopolysaccharide types. O2a was the most common type (49.2%, *n* = 93), followed by O5 (12.7%, *n* = 24), O1 (9.5%, *n* = 18), and O3b (1.6%, *n* = 3). In ST11 isolates, 55.4% (*n* = 93) were typed O2a, 14.3% (*n* = 24) were O5, and the rest were unclassified. All of the ST15 and ST2237 isolates were of the O1 type (Fig. 1; Table S6).

### Reconstruction of phylogenetics.

**FIG 2:**
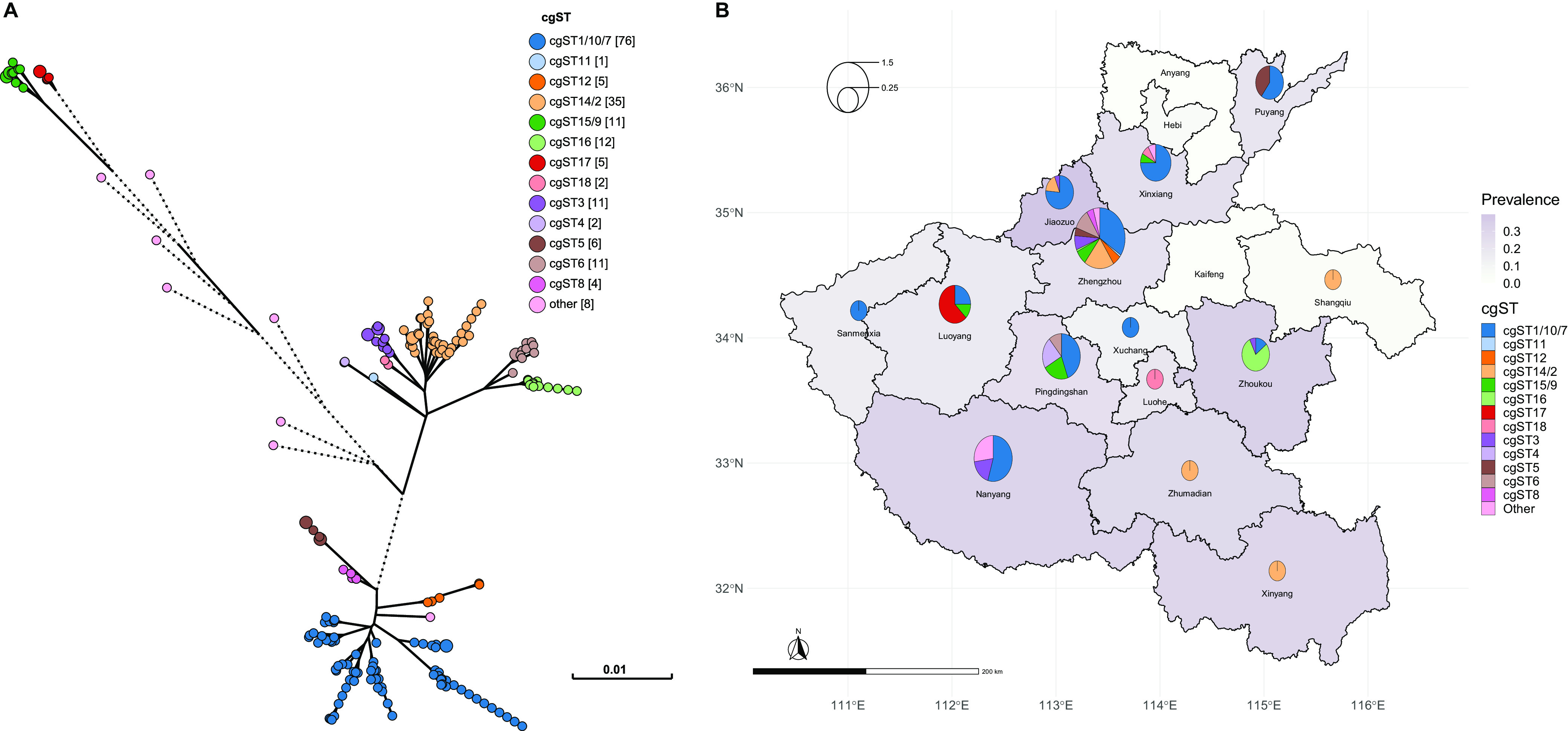
Phylogenetic relationship and geographic distribution. (A) Phylogenetic relationship of the isolates. Circles represent isolates and are colored according to their cgST groups. Numbers in square brackets are the count of isolates in each group. Edges longer than 0.01 are shortened and shown in dotted lines. (B) Choropleth map of the geographic distribution of the isolates. Regions are colored to indicate the prevalence of CRKP in the survey. The size of the pie charts is scaled according to the Shannon diversity index. Each slice in the pie charts represents a cgST detected in the corresponding region and is scaled to indicate the number of isolates.

The phylogeny of the isolates was reconstructed based on the allelic differences of the core genes. ST11 isolates formed very closely related lineages that grouped into two clades, while isolates that belonged to other sequence types were at a greater genetic distance. The geographical distribution of the isolates showed a great disparity in prevalence and diversity in different regions of the province. Higher prevalence and diversity were found in the northwestern cities. Specifically, CRKP was found to be most prevalent in Jiaozuo, and the population was most diverse in Zhengzhou, the capital city that hosts multiple central hospitals and serves as one of the top destinations for hospital transfers (Fig. 2).

### Multisite colonization.

In 59 patients, we obtained CRKP isolates from both the respiratory tract and the gut. With the core genome sequence typing results, we determined that in 44 patients, the duo isolates (or trio in one case) belonged to the same monophyletic clades and hence were likely originating from single colonization events. To further validate that these isolates were monoclonal, we performed pairwise whole-genome alignments and variant calling. It was found that in these 44 patients, the genomes shared 99.996% bases in 99.56% of the genome on average. These genomes also had a low number of single nucleotide variations (SNVs) between them, significantly lower than that of nonmonoclonal isolates (median, 13 versus 327; Wilcoxon test, *P* < 0.0001) (Table S7). We compared the ratio of monoclonal isolates with the expected ratio of random colonization events and showed that intestinal carriage was associated with colonization in the respiratory tract (odds ratio = 10.80, *P* < 0.0001) (Table S8).

### Antimicrobial resistance.

**FIG 3:**
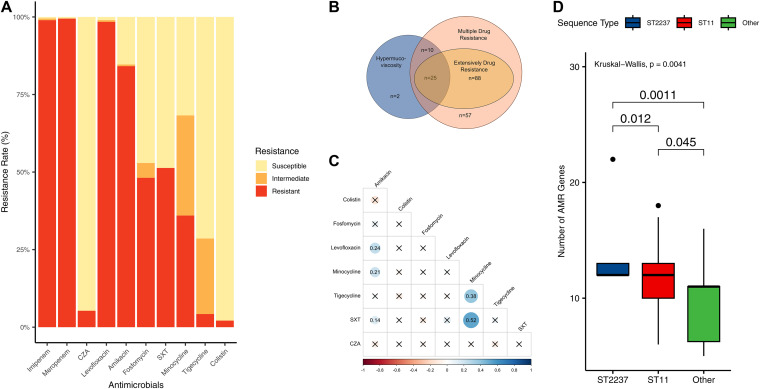
Antimicrobial resistance and hypermucoviscosity characteristics. (A) Resistance rate of the antimicrobials. (B) Venn graph showing the number of multidrug-resistant, extensively drug-resistant, and hypermucoviscous isolates. (C) Cross-resistance between antimicrobials. Imipenem and meropenem are excluded, as the isolates have been selectively cultured by meropenem. (D) Difference in the number of AMR genes carried by ST2237, ST11, and other sequence types.

All isolates were confirmed by MIC tests to be resistant to carbapenem. Also, most of the isolates were also resistant to amikacin (84.7%, *n* = 160) and levofloxacin (98.9%, *n* = 187) but susceptible to ceftazidime-avibactam (94.7%, *n* = 179) and colistin (97.9%, *n* = 185). Fosfomycin and SXT were effective against roughly half of the isolates (47.1%, *n* = 89, and 48.7%, *n* = 92 susceptible, respectively). Resistance to tigecycline and minocycline was dose dependent, with tigecycline being resisted in fewer cases than minocycline (4.2%, *n* = 8, and 36.0%, *n* = 68 resistant; 24.3%, *n* = 46, and 32.3%, *n* = 61 intermediate, respectively). Most (95.2%, *n* = 180) of the isolates showed multiple drug resistance (MDR), defined as being nonsusceptible to >2 classes of antimicrobials other than carbapenem, and 59.8% (*n* = 113) exhibited extensive drug resistance (XDR), defined as being nonsusceptible to all but two or fewer antimicrobial categories (41). Cross-resistance between the antimicrobials was evident. Isolates exhibiting resistance to amikacin were also likely to be resistant to levofloxacin (*r* = 0.24, *P* = 0.000757), minocycline (*r* = 0.21, *P* = 0.00307), and SXT (*r* = 0.14, *P* = 0.0489), while minocycline resistance was correlated with tigecycline (*r* = 0.38, *P* < 0.0001) and SXT (*r* = 0.52, *P* < 0.0001) (Table S9; Fig. 3).

**FIG 4:**
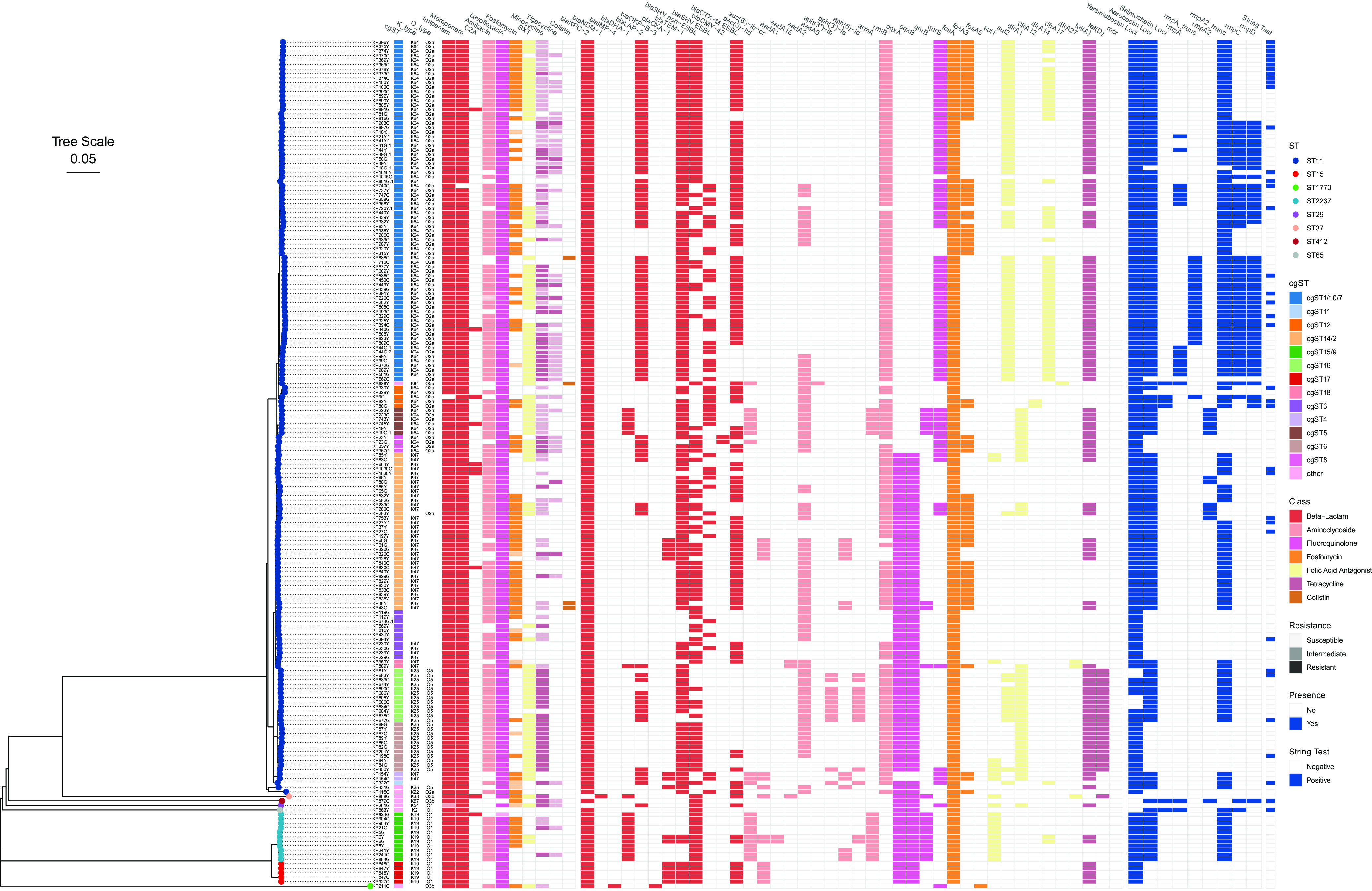
Phenotype and genotype profiles of antimicrobial resistance and hypermucoviscosity. The leftmost panel is the core genome phylogenetic tree of the 189 isolates, with leaves colored according to the ST lineages. The cgST, K-type, and O-type are listed next to each isolate. Types with mismatches or uncertainty are not shown. Resistance to each of the antimicrobials tested is shown in the next panel, colored by the class of the antimicrobials and shaded lightly if the isolate shows intermediate resistance. The presence of genes is shown in the same color as their corresponding drug class. Hypermucoviscosity phenotype, as determined in the string test, and genotype are shown in deep blue boxes. A filled box indicates a positive test result or that a gene is present and vice versa.

All 189 isolates were carbapenemase producers. We detected the presence of carbapenemase-encoding genes (*bla*_KPC_, *bla*_NDM_, *bla*_IMP_), *AmpC* beta-lactamase (Ambler class C)-encoding genes (*bla*_DHA_, and *bla*_CMY_), oxacillinase (Ambler class D)-encoding genes (*bla*_OXA_), and extended-spectrum beta-lactamase (ESBL)-encoding alleles (*bla*_SHV-12_, *bla*_CTX-M-14_, *bla*_CTX-M-15_, *bla*_CTX-M-65_, etc.). Both strains of ST2237, the newly identified sequence type in Henan, and that of ST11, the predominant type, carried more AMR genes, with statistically significant differences from other types (Fig. 3). The isolates predominantly possessed *bla*_KPC-2_ (187/189, 98.9%). Metallo-beta-lactamase (MBL) genes were detected at a relatively low frequency: isolate KP868G was the only one harboring *bla*_NDM_, and the K. quasipneumoniae isolate KP211G was the only *bla*_IMP_ carrier. Also, 79.8% (134/168) of the ST11 isolates, 100% (5/5) of the ST15 isolates, and 18.2% (2/11) of the ST2237 isolates, totaling 75.7% (143/189), were found to be coharboring CTX-M or SHV ESBL genes (Table S10; Fig. 4).

Only 105 out of the 187 isolates resistant to levofloxacin harbored *qnr* genes, and there was no statistically significant correlation. Further analysis of the quinolone resistance-determining region (QRDR) revealed that nearly all of them (*n* = 185) carried *gyrA* (S83I and D87G) and *parC* (S80I) mutations, which was significantly correlated with levofloxacin resistance (*r* = 0.703, *P* < 0.0001). SXT resistance was strongly correlated with the presence of *dfrA* genes (*r* = 0.852, *P* < 0.0001) but also showed a weaker correlation with the presence of *sul* genes (*r* = 0.301, *P* < 0.0001). Fosfomycin resistance was associated with *fosA3* (*r* = 0.624, *P* < 0.0001), and resistance to tetracyclines, including minocycline and tigecycline, was associated with *tet* genes (*r* = 0.560, *P* < 0.0001, and *r* = 0.236, *P* = 0.001078, respectively) (Table S10; Fig. 4).

Notably, despite 4 isolates being found to be resistant to colistin, we did not find any member from the colistin resistance gene family *mcr* or mutations in *pmrB* in any of the genomes. Two of them, however, carried a truncated copy of the *mgrB* gene caused by the insertion of an IS5-like insertion sequence, which has been shown to confer colistin resistance (42, 43). Of the 10 isolates exhibiting resistance to CZA, KP868G was the *bla*NDM-producer, and the other 9 were carrying *bla*SHV ESBLs and/or mutations in OmpK35 and Omp36 osmoporins (Table S11). Regularized regression with cross-validation revealed that *bla*_CTX-M-27_, *bla*_CTM-M-65_, *bla*_DHA-1_, *bla*_NDM-1_, *bla*_SHV-119_, *bla*_SHV-12_, *bla*_SHV-172_, and mutation in OmpK35 and OmpK36 all contributed positively to the resistance of CZA.

### Hypermucoviscosity.

We determined the hypermucoviscosity phenotype of the isolates by string tests and obtained an estimation of the prevalence at 19.6% (*n* = 37). The phenotype was significantly associated with ST11, ST421, and ST65 sequence types (*P* < 0.0001). Siderophore production genes, including *ybt* for yersiniabactin, *iuc* for aerobactin, and *iro* for salmochelin (including their associated regulators like *iutA*), were detected in 94.7% (*n* = 179), 79.9% (*n* = 151), and 3.2% (*n* = 6), respectively, of the isolates. In addition, the *cps* loci genes, including *rmpA*, *rmpA2*, *rmpC*, and *rmpD*, were found in 19.6% (*n* = 37) of the isolates. Notably, rmpA2 was found often in truncated forms (73.0%, *n* = 138), regardless of the presence of other *cps* genes. Other virulence factors that had not been reported to be hvKP associated were also detected, mainly including genes involved in capsule synthesis, fimbriae regulation, and enterotoxin production (Table S10). In the regularized logistic regression, the presence of aerobactin loci, salmochelin loci, and truncated *rmpA2*, *fimB*, *manC*, *senB*, and *tle1* was found to be the primary hypermucoviscosity-associated factors. Of them, the presence of aerobactin loci and *manC* were also found to be positively correlated with the capsular type K64 (*r* = 0.32, *P* < 0.0001, and *r* = 0.72, *P* < 0.0001, respectively).

Of particular interest, one of the hospitals hosted 11 patients from whom 14 isolates were recovered. Twelve of the isolates manifested a hypermucoviscosity phenotype, and all but one of them were classified as cgST1/10/7 (ST11) strain with capsule type K65 and lipopolysaccharide type O2a. These isolates also shared a similar virulome, harboring *ybt*, *iuc*, and *rmp* genes with nearly identical alleles. This evidence strongly suggested that these isolates were of a CRKP strain that acquired hypervirulence and spread within the hospital in an outbreak event.

## DISCUSSION

In this multicenter cross-sectional study, comprehensive large-scale surveillance enabled us to acquire an extensive collection that reflected the prevalence and diversity of CRKP in an epidemic region. The high prevalence and geographical diversity suggested a widespread antimicrobial resistance mixed with endemics of locally specific strains, both of which contribute to an alarming and unbalanced burden on public health. In a recent smaller-scale clinical survey of CRKP from bacteremia in China, ST11, ST45, ST15, and ST290 were found to be the predominant lineages (44). Our data expanded the evidence base to the conclusion that ST11-KL64 and ST11-KL47 are the predominant types of CRKP in China (35, 45). Meanwhile, our finding of the increased frequency of ST2237 and the discovery of the new lineage, ST587, called for attention of disease control and stressed the importance of continued monitoring and surveillance. We also identified a multidrug-resistant K. quasipneumoniae isolate which carried the metallo-beta-lactamase *bla*_IMP-4_, but no hypervirulent factors. The importance of K. quasipneumoniae in clinical settings has recently been recognized, but its clinical and molecular characteristics remained largely unknown (46–48). These findings warrant further investigation into whether K. quasipneumoniae plays a role in the transmission of antimicrobial resistance genes to more pathogenic K. pneumoniae strains.

K. pneumoniae, as an opportunistic pathogen, is part of the normal gut microbiota, with a rate of intestinal colonization estimated at 6% (19). A recent study showed that WGS-confirmed nosocomial transmission was implicated in just 10% of cases but strongly associated with ESBLs (49). Our study demonstrated that in most patients, isolates from the respiratory tract and the gut were of the same origin, thereby supporting the theory that gastrointestinal carriage serves as a reservoir of respiratory infection. On the other hand, the nosocomial transmission of hypervirulent CRKP strains was also evident in our study, indicating that both sources of infections in the ICU should not be underestimated.

In accordance with previous reports, we found CRKP isolates in the study exhibited multiple drug resistance and cross-resistance, yet colistin and ceftazidime-avibactam remained the most effective. KPC, ESBL, and NDM-positive K. pneumoniae were characterized as among the predominant causes of carbapenem resistance, although in different strains in surveillance conducted in China, Singapore, South Korea, Italy, Australia, and the United States (50–55). In this study, we found that *bla*_KPC-2_ was the most common carbapenemase type, but ESBLs were also prevalent, in accordance with other studies (56, 57). In a previous study, *bla*_NDM_-positive K. pneumoniae was recovered at a rate of 8.3% (4/48) in Henan (58). In another recent survey in Hong Kong, the detection rate of the *mcr* gene using WGS was 5.3%, and 50.9% were ESBL producers (59). We observed no *mcr* carriers and a much lower *bla*_NDM_ prevalence. It has been reported that MBL production, *bla*_KPC-2_ point mutation (D179Y), and excessive expression of KPC could result in CZA resistance, which may be alleviated by increasing the amount of avibactam to CZA (60). Our study partially agreed with this conclusion but also pointed to the association of CZA resistance with mutations in *bla*SHV and/or OmpK35 and OmpK36 osmoporins. A recent study concluded that *mgrB* alterations were the main reason for colistin resistance and resistant strains were polyclonal (61). Our data supported the role of altered *mgrB* in colistin resistance but also highlighted isolates whose colistin resistance remained unexplained, inviting further investigation.

Hypervirulent markers were carried by a remarkable proportion of the isolates. Consistent with previous studies (30), we found the presence of aerobactin and *rmpA*-related genes correlated with hypervirulence, and we also identified other potential markers. The overall high prevalence of hypermucoviscosity phenotype in our CRKP collection again brings to attention the worrying evidence of AMR-virulence convergence, which has previously been reported in Asia (33, 62). As such convergence is found at increasing frequency, it is imperative to prevent the spread of the difficult-to-treat hypervirulent CRKP strains through improved infection control measures (36).

It should be noted that in this study, expressivity and penetrance of AMR genes were only speculative and could not be accurately estimated. Additionally, in the statistical analyses, correlated variables should not be interpreted as being involved in any molecular mechanisms of casual relationships without further evidence. Another limitation of this study is that we did not analyze the genomic characteristics in combination with patient outcomes.

### Conclusions.

Our survey revealed that the prevalence of CRKP per ICU patient in Henan, China, was 20.9%, and the predominant sequence type was ST11 (88.9%). We found ST2237 strains at an increased frequency over previous studies and a new ST587 strain. We studied the geographical distribution and phylogenetics of prevailing lineages, and our results supported that gastrointestinal carriage serves as a reservoir of respiratory infection. We also found 95.2% of the isolates were multiple drug resistant and 59.8% were extensively resistant, in accordance with the high carriage rate of *bla*_KPC-2_ (98.9%) and ESBLs (75.7%). We determined that aerobactin and salmochelin sequence types, among others, were predictors of hypermucoviscosity, and our findings provided evidence for a nosocomial outbreak event of an ST11-K65-O2a CRKP strain that acquired hypervirulence.

## MATERIALS AND METHODS

### Isolate collection and phenotypic characterization.

As part of a carbapenem-resistant organism screening program, we conducted a multicenter cross-sectional study of CRKP colonization in ICU inpatients in 78 hospitals in Henan Province, China. Specimens of bacteria in the respiratory tract and the gut, including throat swabs, nasopharynx swabs, sputum, rectal swabs, and ostomy swabs, were collected from all the ICU inpatients in the participating hospitals on 10 March 2021. Specimens were cultured at 37°C for ~6 to 8 h before being transferred to the selective culture medium containing meropenem (4 μg/mL) and cultured at 37°C overnight, which allowed the selection of carbapenem-resistant organisms (63). Positive cultures were incubated on MacConkey agar plates at 37°C for 24 h. Isolates were picked and identified using MALDI-TOF MS and tested by Kirby-Bauer disk diffusion test to confirm resistance to meropenem. K. pneumoniae strains ATCC BAA-1705 (carbapenem resistant) and ATCC BAA-1706 (carbapenem susceptible) were used as positive and negative controls, respectively. We downsampled the isolates for further investigation through stratified random sampling. *In vitro* antimicrobial susceptibility was determined by the automated antibiotic susceptibility testing system Phoenix 100 (BD, Sparks, MD). The panel of antimicrobials included two carbapenems (imipenem and meropenem), an aminoglycoside (amikacin), a cyclic lipopeptide (colistin), a phosphoenolpyruvate analog (fosfomycin), a fluoroquinolone (levofloxacin), a synergistic folate antagonist combination (SXT), a cephalosporin combined with beta-lactamase inhibitor (CZA), and two tetracyclines (minocycline and tigecycline). The Clinical and Laboratory Standards Institute (CLSI)-recommended breakpoints of MICs were used. The string test was performed on each of the CRKP isolates as previously described (64). This study was conducted in accordance with the National Guide to Clinical Laboratory Procedures (65).

### Whole-genome sequencing, assembly, and annotation.

The isolates were sequenced on the Illumina platform to 150-bp paired-end reads. The raw reads were subjected to quality control using fastp version 0.23.2 (66). Reads that were too short (<50 bp) or with a quality score of <15 in over 40% of the bases were discarded. *De novo* genome assembly was performed using SPAdes version 3.15.4 (67). The assembled genomes were quality checked with BUSCO version 5.2.2 and CheckM version 1.1.3 (68–70). Contigs were removed if they were shorter than 200 bp or had a mean depth smaller than 5 per base. Species identification was done by comparing the assembly using Mash distance with reference sequences (71). Assembled genomes were annotated using Prokka version 1.14.5 (72). Antibiotic resistance genes were identified using ResFinder version 4.0, and virulence genes were identified by aligning the reads with records in VFDB 2019 (73, 74).

### Pan-genome analyses and sequence typing.

The pan-genome of the K. pneumoniae isolates included in this study was determined using PEPPAN version 1.0.6 (75). Sequence type was defined as an allelic combination of 7 housekeeping genes determined by *in silico* multilocus sequence typing (MLST) using BacWGSTdb (76). The analysis was based on the established K. pneumoniae MLST scheme described in the K. pneumoniae BIGSdb hosted at the Pasteur Institute, including *gapA*, *infB*, *mdh*, *pgi*, *phoE*, *rpoB*, and *tonB* (77). Clonal group (CG) was defined as related STs differing only in one or two alleles (7). Core genome sequence typing was conducted using SeqSphere+ version 8.2.0 (Ridom GmbH, Germany), using the Klebsiella pneumoniae *sensu lato* complex reference schema. Genetic distances between genomes were calculated by comparing the cgMLST allelic profiles. Single-linkage hierarchical clustering was used to assign the genomes to clusters with stable nomenclature, which we called core genome sequence types (cgSTs). Kleborate version 2.2.0 with its Kaptive module was used to predict K-antigen and O-antigen types, as well as virulence gene locus sequence types (13, 78, 79).

### Whole-genome alignment, variant calling, population structure, and phylogenetics.

Whole-genome alignment was performed to study the similarities and differences of the putative monoclonal genomes. NUCmer version 3.1 and DNAdiff version 1.3 from the MUMMER package (80) were used for doing the alignment and calculating the summary statistics, respectively. Variant calling was performed with Snippy version 4.6.0. Population structure and geographical distribution of different sequence types were analyzed with R software version 4.1.2 (81). Shannon’s diversity index at different geographical locations was calculated with the vegan package, and the map was drawn with maptools, ggplot2, and scatterpie packages (82–85). The cgMLST trees were inferred with the neighbor-joining algorithm and visualized with GrapeTree version 1.5.0 (86). The rooted tree used isolate KP221G as an outgroup and was drawn using the ggtree package (87–89).

### Statistical analysis.

Statistical analysis was performed with R software version 4.1.2. The association between dichotomized sources of bacteria, i.e., swab types, and categorical sequence typing was inferred by Pearson’s χ^2^ test for independence. Pearson’s correlation coefficients were used to evaluate the strength and direction of the relationship between genetic features of interest and antimicrobial resistance. Kruskal-Wallis test and Mann-Whitney-Wilcoxon test were used to compare the differences in the number of SNVs and the number of AMR genes. Regularized logistic regression, with cross-validation, was used to perform variable selection, examine the combined effect of relevant genetic features on phenotypes, and identify predictive markers. We applied the elastic net algorithm, which combines both penalties in LASSO and ridge regression, in the regression analysis (90, 91). Eligibility for model inclusion of the genetic features was based on biological plausibility and previous studies (30, 92). Tenfold cross-validation was used for the analysis of virulence factors, and the leave-one-out strategy was applied for the analysis of CZA resistance due to an insufficient number of observations (*n* = 10). All *P* values of <0.05 were considered statistically significant, and only *P* values of ≥0.0001 were reported as exact numbers.

### Ethics approval and consent to participate.

This study was approved by the medical ethics committee of Henan Provincial People’s Hospital. Informed consent was obtained from the patients.

### Data availability.

The data supporting the conclusions of this article are included within the article and its additional files. Sequence data supporting the conclusions of this article are available in the GenBank repository, with the BioProject accession number PRJNA870270.
