# Supplementary material for: Genomic Epidemiology and Characterization of Carbapenem-Resistant Klebsiella pneumoniae in ICU Inpatients in Henan Province, China: a Multicenter Cross-Sectional Study
Source: Microbiol Spectr. 2023 May 22;11(3):e04197-22. doi: 10.1128/spectrum.04197-22 (PMC10269698; doi:10.1128/spectrum.04197-22)
Supplement: Supplemental file 1 — Supplemental material. Download spectrum.04197-22-s0001.pdf, PDF file, 0.2 MB [file spectrum.04197-22-s0001.pdf]

Supplemental Material

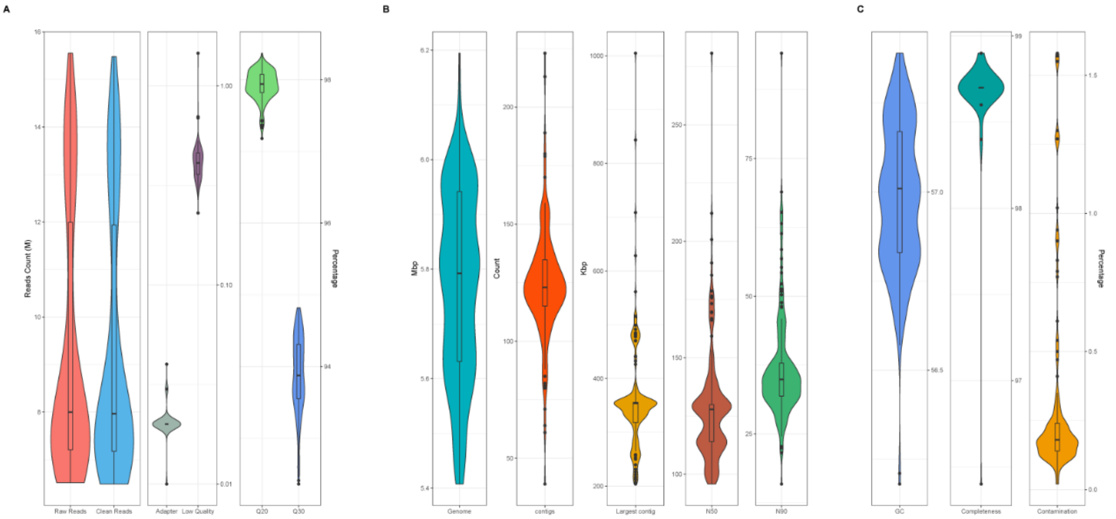

Figure S1 Sequencing and Assembly Statistics. A Distribution of raw reads, clean reads, adapter ratio, low quality reads ratio, Q20, and Q30 percentages. **B** Distribution of assembled genome lengths, number of contigs, size of the largest contig, N50 and N90 values. **C** Distribution of GC content, completeness and contamination ratio of the assemblies.

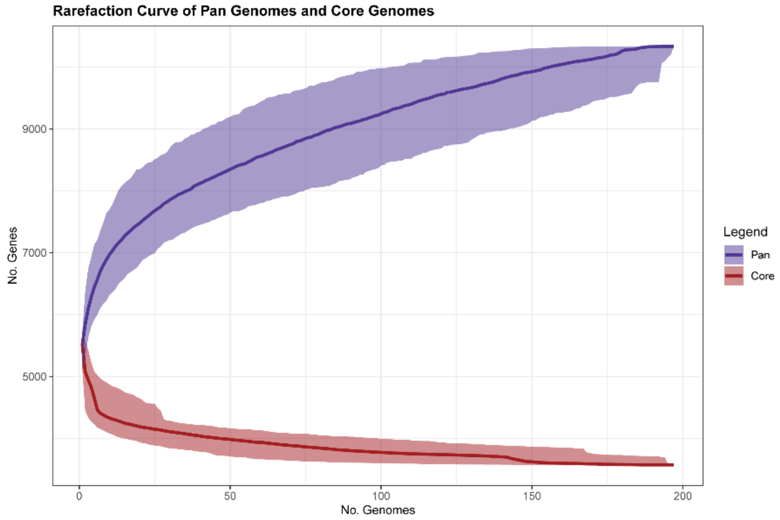

Figure S2 Rarefaction Curve of Core and Pan Genes. Number of core and pan genes is plotted against the number of genomes include. Core genes are colored in red and pan genes purple. The shaded area indicates the 95% confidence interval.
